# Early Events following Experimental Infection with Peste-Des-Petits Ruminants Virus Suggest Immune Cell Targeting

**DOI:** 10.1371/journal.pone.0055830

**Published:** 2013-02-13

**Authors:** Robert A. Pope, Satya Parida, Dalan Bailey, Joe Brownlie, Thomas Barrett, Ashley C. Banyard

**Affiliations:** 1 The Pirbright Institute, Pirbright, Woking, Surrey, United Kingdom; 2 Royal Veterinary College, Hatfield, Hertfordshire, United Kingdom; Virginia Polytechnic Institute and State University, United States of America

## Abstract

Peste-des-petits ruminants virus (PPRV) is a viral pathogen that causes a devastating plague of small ruminants. PPRV is an economically significant disease that continues to be a major obstacle to the development of sustainable agriculture across the developing world. The current understanding of PPRV pathogenesis has been heavily assumed from the closely related rinderpest virus (RPV) and other morbillivirus infections alongside data derived from field outbreaks. There have been few studies reported that have focused on the pathogenesis of PPRV and very little is known about the processes underlying the early stages of infection. In the present study, 15 goats were challenged by the intranasal route with a virulent PPRV isolate, Côte d’Ivoire ’89 (CI/89) and sacrificed at strategically defined time-points post infection to enable pre- and post-mortem sampling. This approach enabled precise monitoring of the progress and distribution of virus throughout the infection from the time of challenge, through peak viraemia and into a period of convalescence. Observations were then related to findings of previous field studies and experimental models of PPRV to develop a clinical scoring system for PPRV. Importantly, histopathological investigations demonstrated that the initial site for virus replication is not within the epithelial cells of the respiratory mucosa, as has been previously reported, but is within the tonsillar tissue and lymph nodes draining the site of inoculation. We propose that virus is taken up by immune cells within the respiratory mucosa which then transport virus to lymphoid tissues where primary virus replication occurs, and from where virus enters circulation. Based on these findings we propose a novel clinical scoring methodology for PPRV pathogenesis and suggest a fundamental shift away from the conventional model of PPRV pathogenesis.

## Introduction

Peste-des-petits ruminants virus (PPRV) is the causative agent of an economically significant disease of small ruminants, peste-des-petits ruminants (PPR). PPR causes significant losses due to high morbidity and high mortality rates, with the latter occasionally approaching 90–100% in naïve populations, dropping to nearer 20% in endemic areas [1]. The highly contagious nature of the virus and the movement of animals through trade or nomadic lifestyles, create a serious trans-boundary problem, inhibiting trade and heightening economic losses in affected areas, where small ruminants predominate in the livestock population (reviewed by [2]). PPR is included by the OIE (Office International d’Epizooties) in its list of notifiable animal diseases and is now endemic in the majority of Saharan and sub-Saharan Africa, Turkey, the Middle East and the Indian sub-continent [2], [3]. Recent isolations of PPRV have been recorded in Morocco, China, Algeria; Tajikistan, Sierra Leone and the Democratic Republic of Congo [4]–[9].

PPRV is classified within the Order *Mononegavirales*, family *Paramyxoviridae*, sub-family *Paramyxovirinae*, genus *Morbillivirus*, and shares both structural and clinicopathogenic characteristics with the other members of the genus; *Measles Virus* (MV), *Rinderpest Virus* (RPV), *Canine Distemper Virus* (CDV), *Phocine Distemper Virus* (PDV), *Porpoise Morbillivirus* (PMV), *Dolphin Morbillivirus* (DMV) and *Feline Morbillivirus* (FMV) [10]–[13]. Further to these well defined members of the genus, numerous novel morbillivirus-like pathogens have been discovered in both bat and rodent populations [14]. While there have been some experimental analyses of the pathogenesis of PPRV [15], [16] most of the data used to create the current understanding of PPR infection is derived from natural outbreaks in the field [17]–[24]. These studies have demonstrated that the clinical disease caused by PPRV strongly resembles those caused by the other morbilliviruses and in particular that seen with the infection of large ruminant species with RPV, which has recently been globally eradicated [25].

PPRV is both lympho- and epitheliotrophic and infection typically results in pyrexia, conjunctivitis, rhinotracheitis and ulcerative stomatitis, gastroenteritis and in severe cases, pneumonia [26]. The disease may have an acute and severe course in goats and sheep [27], but is not considered to be pathogenic for large domestic ruminants [28] although several large ruminant species have been found to be seropositive when present during an outbreak in surrounding small ruminants [29]. Interestingly, on one occasion a clinical case of PPRV infection was reported following experimental inoculation of calves [30] and a further report describes an outbreak of clinical disease in buffalo caused by PPRV [31]. Infection of wildlife species appears to be less of a feature than in the epidemiology of RPV, but fatal, natural infections of gazelle, gemsbok, bharals, ibex and bison have been reported [18], [31]–[34] (reviewed in [2]).

Often, where epidemics have been reported in the field, clear conclusions from pathogenesis studies have been hampered by lack of control materials, infection with non-standardised doses of virus via animal to animal contact and the possibility of multiple infection routes. Alongside this, considerations of breed susceptibility, immunocompetence of hosts, and existing parasitic infections that might be exacerbated by an immunosuppressive agent such as PPRV are largely undefined. One experimental study attempted to mimic natural infection by using an intra-nasal route of infection although events during the early stages of infection were not reported alongside virus distribution during late stage disease [16]. For other morbilliviruses, studies have investigated the basic biological mechanisms of entry and dissemination for both CDV [35], [36] and MV [37]. Recent studies with MV have highlighted the role of alveolar macrophages and dendritic cells in infection and have suggested an immune cell driven movement of MV from initial sites of infection within alveolar mononuclear cells to lymphoid organs for virus amplification [38].

In the present study, we report a structured analysis of the pathogenesis of PPRV, concentrating on early events following intranasal inoculation with a field isolate of PPRV. Alongside this we have developed a clinical scoring methodology to enable assessment of infection both in the field and naturally and use data within this study to illustrate our template. In conclusion, we propose a similar mechanism to that suggested for MV, for the early stages of PPRV infection whereby virus infects immune cells within the respiratory mucosa which then migrate to T-cell–rich areas of local lymphoid organs including the tonsil from which virus enters general circulation.

## Results

### Clinical Observations Following Infection with the Côte d’Ivoire ’89 (CI/89) Strain of PPRV

**Figure 1:**
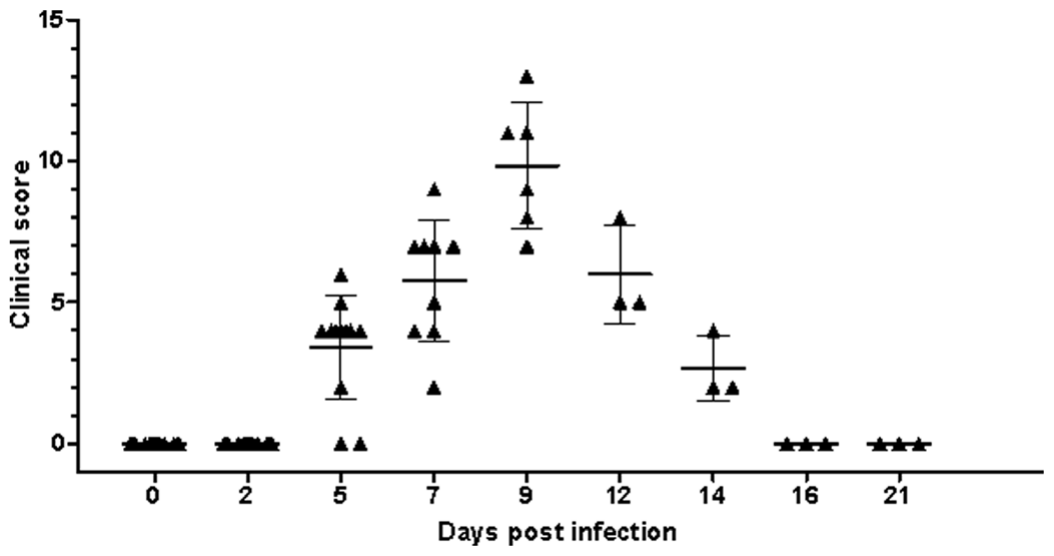
Clinical disease following experimental inoculation. Experimentally inoculated animals were graded by clinical observation for the development of disease consistent with infection with PPRV. Individual animal scores are shown for each sampling point with the standard deviation around the mean being illustrated.

Following infection, clinical signs were generally mild with hyperaemia of the nasal mucosa and a scanty clear nasal discharge. This study, and previous experimental inoculations, were used to develop a clinical score sheet suitable for grading from mild to severe PPRV infections during pathogenesis studies. All animals were scored clinically according to the clinical score sheet (Table 1). Peak clinical observations along with time of euthanasia are detailed in Figure 1. Clearly, peak clinical disease was seen at 9 dpi with remaining animals clearing infection and going on to convalesce (n = 3).

**Table 1:** Clinical score sheet for assessment of animals infected with PPRV.

Clinicalscore	General signs	Pyrexic response	Ocular/nasal discharge	Facial mucosal lesions	Faeces	Respiratory symptoms
**0**	Normal	<39.5°C	None	None	Normal	Normal respiration rate (Sheep:15–40*; Goats: 10–30**)
**1**	Mildly inactive	>39.5°C but <40°C	Watery oculardischarge	Congested oronasal mucosaand buccal papillae	Soft	Slight tachypnoea
**2**	Mildly inactive anddepressed, mildinappetance	>40°C but <41°C	Watery to mucoid oculonasal discharge: reddened eyes andmild conjunctivitis	Pin-prick lesions within buccalcavity, with some becomingmore extensive	Runny	Tachypnoea/mild cough
**3**	Inactive, apathetic,restless andanorexic	>41°C or >39.5°Cfor >5days	Mucopurulent nasaldischarge and/or severeconjunctivitis with mucopurulent oculardischarge	Clear erosive lesions onoronasal mucosae; severelycongested/oedematousbuccal papillae	Frank diarrhoea	Tachypnoea and dyspnoea/coughing present
**4**	Severe depression,unable to stand,extreme lethargy,dehydration	>41°C or >39.5°Cfor >5 daysfollowed byrapid fall intemperature(<38°C*)	Mucopurulent nasaldischarge and severeconjunctivitis withprofuse mucopurulentocular discharge	Severe erosive/ulcerativelesions throughout buccalcavity, nasal mucosa andnares; oedematous lips anderosions on vulval labia	Muco-haemorrhagic diarrhoea	Marked tachypnoea/dyspnoea/cough

When animal reaches a score of 20 they need to be killed on ethical grounds. The decision to euthanase would be based on the following criteria: 1) A score of 4 is achieved in "General Signs“; 2).

A score of 3 is achieved in "General Signs" for 2 complete, consecutive days and a score of 10 or greater is achieved in other categories; 3) A.

score of 2 is achieved in "General Signs" for 2 complete, consecutive days and a score of 15 or greater is achieved in other categories. (*Hecker.

(1983) The sheep as an experimental animal. Academic Press, London; **Smith and Sherman (2009) Goat Medicine, Wiley-Blackwell. Ames,

Iowa, USA).

**Figure 2:**
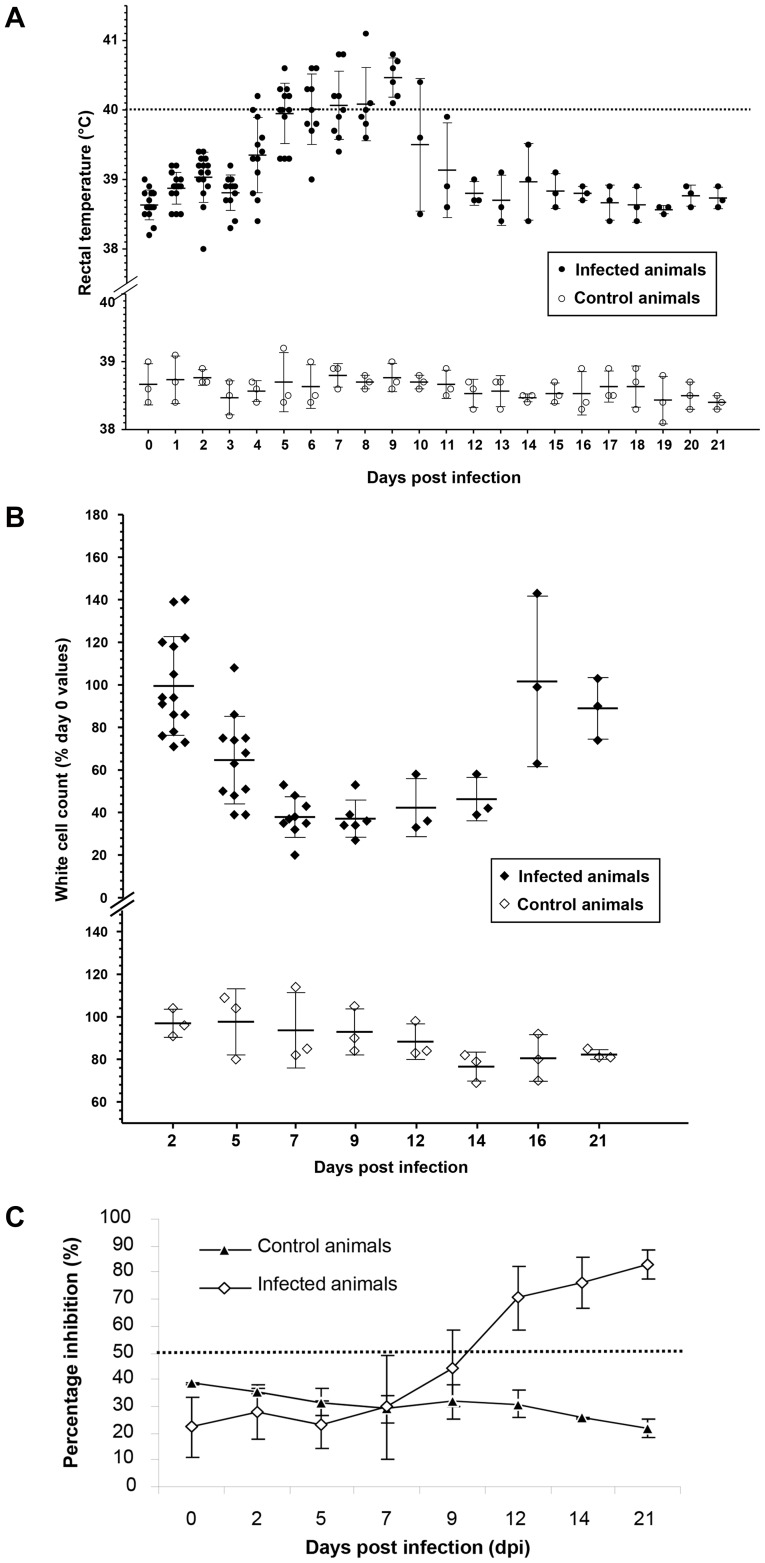
Clinical monitoring and serological response following infection with the PPRV CI/89 strain. a) Average rectal temperatures of goats infected with PPRV CI/89. The dashed dotted line indicates the temperature cut-off value above which animals were considered pyrexic (39.5°C). Average values are plotted for infected (n = 15) against control (n = 3) animals with standard deviation around the mean being shown. Animal numbers reduce in line with the staggered euthanasia performed; **b)** Infection induced leucopaenia following infection with PPRV CI/89. PBL counts are expressed as a percentage change from the three-day, pre-challenge average of each goat’s PBL counts. Mean values and standard deviation is shown at each time point (Day 0 PBL counts taken as 100%); **c)** Serological responses following inoculation with the PPRV CI/89 strain in the animals that remained at the end of the study (n = 3). The PI cut off value for seropositivity is denoted by a dotted line.

No visible clinical features were noted on days 2 or 5 post infection although some animals were pyrexic as early as 4 dpi (n = 5; 41.7%) and so had achieved a clinical score according to the clinical score sheet (Figure 1 and Figure 2A). By day 7 animals had developed reddening of the facial mucosae, often accompanied by mild/moderate sero-mucoid/muco-purulent oculonasal discharges by day 9 (n = 6; 100%). Some animals developed mild facial mucosal erosions from days 9–12, although these had resolved by 16 dpi (n = 3; 66.67%). Some goats exhibited signs of inappetance and developed a transient diarrhoea (n = 3; 50%) from days 9 to 12 post inoculation. The clinical condition of all animals had improved by 14 dpi and all were clinically normal at 16 dpi. None of the control goats (n = 3) displayed any clinical signs during the experimental period.

### Pyrexia and Leucopaenia

All virus inoculated goats became pyrexic (defined as a temperature greater than 40°C- normal temperature considered to be 38.6°C) between 4 and 9 dpi (Average 5 days) (Figure 2A). Pyrexic responses were seen from 4 dpi and, following development, lasted for 6 to 7 days for those animals that completed the study (n = 3) with one animal having a shorter (3 day) pyrexic period. In all cases, all goats that developed a pyrexic response recorded temperatures of greater than 39.5°C. Once rectal temperatures had reached a peak (41.1°C; day 8) they gradually returned to within the normal range in the non-euthanased animals (Figure 2A). The control group temperatures never exceeded 39.3°C (Peak average value: 39.0; n = 6) at any point during the trial period (Figure 2A).

All inoculated goats became leucopaenic with PBL numbers dropping below an accepted reference range of 4–13×10^3^ cells/µl [39] during the experimental period. By 7 dpi, the average PBL count of the entire challenge group had reached approximately 40–50% of the pre-inoculation levels (Figure 2B). PBL counts in animals that survived to the end of the experimentation started to increase from 12 dpi and had normalised by 16 dpi. The control group maintained circulating PBL numbers within the range defined above for the duration of the experiment (Figure 2B).

### Serological Response to Infection

Antibody responses following infection were measured in those animals that completed the study (n = 3) using the competitive ELISA based on the H protein as described previously [40]. A percentage inhibition (PI) of >50% was defined as a positive result [41]. None of the animals had PPRV specific antibodies on day 0. All animals that survived the infection were seropositive by 12 dpi, with one animal seroconverting by 9 dpi. Serological responses continued to increase to >80% at 21 dpi in the animals that remained until the end of the study (Figure 2C).

### Detection of Viral Nucleic Acid and Virus Isolation

All samples were assessed for the presence of viral nucleic acid by both first and second round nested RT-PCR. First round RT-PCR was able to detect viral nucleic acid within RNA extracted from eye swabs but not PBLs at 5 dpi. By day 7, all eye swab material was positive for viral RNA but only 44.4% of PBLs were positive by first round PCR. However, analysis of first round PCR products using a nested second round PCR was able to detect viral nucleic acid in all PBLs and all eye swab samples at both days 5 and 7. Results from both first and second round PCRs are detailed in Table 2. Viral nucleic acid was detected in eye swabs and PBLs of animals until the termination of the study, 21 dpi. No viral RNA was detected in any of the samples taken from uninfected control animals that were housed separately but within the same air space as the infected animals. Attempts to isolate live virus from PBLs were unsuccessful, however, PPRV was isolated from eye swab samples on both 7 (n = 2; 66.67%) and 9 (n = 3; 100%) dpi.

**Table 2:** Molecular detection of viral nucleic acid by RT-PCR.

Time post-challenge	Sample	No. animals positive for the presence of viral RNA/number (P/T)			
		PPRV Côte d’Ivoire		Control animals	
		F1b–F2d	F1–F2	F1b–F2d	F1–F2
Day 0	PBL	0/15	0/15	0/5	0/5
	Eye swab	0/3	0/3	0/1	0/1
Day 2	PBL	0/15	0/15	0/5	0/5
	Eye swab	0/3	0/3	0/1	0/1
Day 5	PBL	0/12	12/12	0/5	0/5
	Eye swab	3/3	3/3	0/1	0/1
Day 7	PBL	4/9	9/9	0/5	0/5
	Eye swab	3/3	3/3	0/1	0/1
Day 9	PBL	4/6	6/6	0/3	0/3
	Eye swab	3/3	3/3	0/1	0/1
Day 12	PBL	2/3	3/3	0/3	0/3
	Eye swab	2/3	3/3	0/1	0/1
Day 14	PBL	2/3	3/3	0/3	0/3
	Eye swab	3/3	3/3	0/1	0/1
Day 21	PBL	1/3	2/3	0/3	0/3
	Eye swab	2/3	3/3	0/1	0/1

RNA was extracted from eye swabs or PBLs and subjected to RT-PCR. The PCR product obtained by using the diagnostic primer set F1b-F2d was used as template for the nested PCR (primer set F1–F2). P =  Number of animals positive for the presence of viral nucleic acid; T =  Number of animals tested.

### Gross Pathological Observations Following Post-mortem Examination

Prior to euthanasia, each animal was scored according to a clinical score system for PPRV, developed as part of this study. Those animals euthanased showing signs of clinical disease were seen to have mild to moderate mucoid/mucopurulent, nasal/oculonasal discharges, and mild erosions within the nasal cavity. In some cases (n = 4, 66.67%) evidence of increased faecal soiling of the hindquarters suggesting mild diarrhoea was seen (Figure 1). At post mortem examination, in general, mild lymphoid haemorrhages were observed infrequently and were not associated with lymph nodes in particular regions. No significant gross pathological lesions were apparent throughout the digestive, respiratory or genito-urinary tracts, though in the ileum, Peyer’s patches were difficult to identify. Absence of pathological lesions in the lungs was unusual and is discussed.

### Histopathological Assessment of Post-mortem Tissues

All post mortem tissues (List available in Table S1) were assessed using both histopathological (HP) and immunohistochemical (IHC) techniques. There was no evidence of viral antigen in tissues examined until 5 dpi (Table 3 and Tables S2 and S3).

**Table 3:** Antigen detection within lymphoid tissues at different days post inoculation following challenge with the CI/89 strain of PPRV.

		Antigen detection in post mortem tissues		
		Day 5	Day 7	Day 9
**RPLN**	Subcapsular Area	+/++	+++	++
	Follicle/Mantle	0/+	++	+/++
	Germinal Centre	+	++	+
	Paracortex	++/+++	++/+++	+/++
	Medulla	+/++	++	0/+
**MLN**	Subcapsular Area	+	+++	++/+++
	Follicle/Mantle	0	++/+++	+/++
	Germinal Centre	0/+	++	+
	Paracortex	++	++/+++	++
	Medulla	+/++	++	+
**LPSLN**	Subcapsular Area	++	++/+++	++/+++
	Follicle/Mantle	+	+/++	+/++
	Germinal Centre	+	+/++	+
	Paracortex	+/++	++	++
	Medulla	++	+	+
**RPSLN**	Subcapsular Area	+	++	++
	Follicle/Mantle	0	+/++	+/++
	Germinal Centre	0/+	+	+
	Paracortex	++	++	++
	Medulla	++/+	+/++	+
**MSLN**	Subcapsular Area	+	++/+++	+++
	Follicle/Mantle	0/+	+/++	++/+++
	Germinal Centre	0/+	++	++
	Paracortex	+/++	++	++
	Medulla	+	+	+/++
**Tonsil**	Follicle/Mantle	0/+	++/+++	++
	Germinal Centre	0/+	+++	+/++
	Diffuse Lymphoid Tissue	++	+++	++
	Crypt Epithelium	+	++/+++	+++
**Spleen**	PALS	0	+	+/++
	Follicle/Mantle	0	/	/
	Germinal Centre	0	/	/
	Red Pulp	0	0/+	+

Tissues were taken on days 2, 5, 7, 9 and 21 and antigen detection was assessed by immunohistochemistry (IHC). Results for days 2 and 21 are omitted as all tissues analysed were negative for virus antigen. Average immunolabelling grades are given following analysis of tissues from 3 animals euthanased at each timepoint. Grades are formulated on a result of viral antigen density throughout a uniform tissue type. Sections were graded on three separate occasions, without referring to previous recorded results to help standardise the classification. Immunolabelling grades are defined as: 0 =  No immunolabelling seen; + =  Mild immunolabelling; ++ =  Moderate immunolabelling; +++ =  Marked immunolabelling. Intermediate grades exist between the above four categories to give the analysis a greater degree of flexibility. /− Tissue type not present within section.

### Viral Antigen Distribution within Lymphoid Tissues

**Figure 3:**
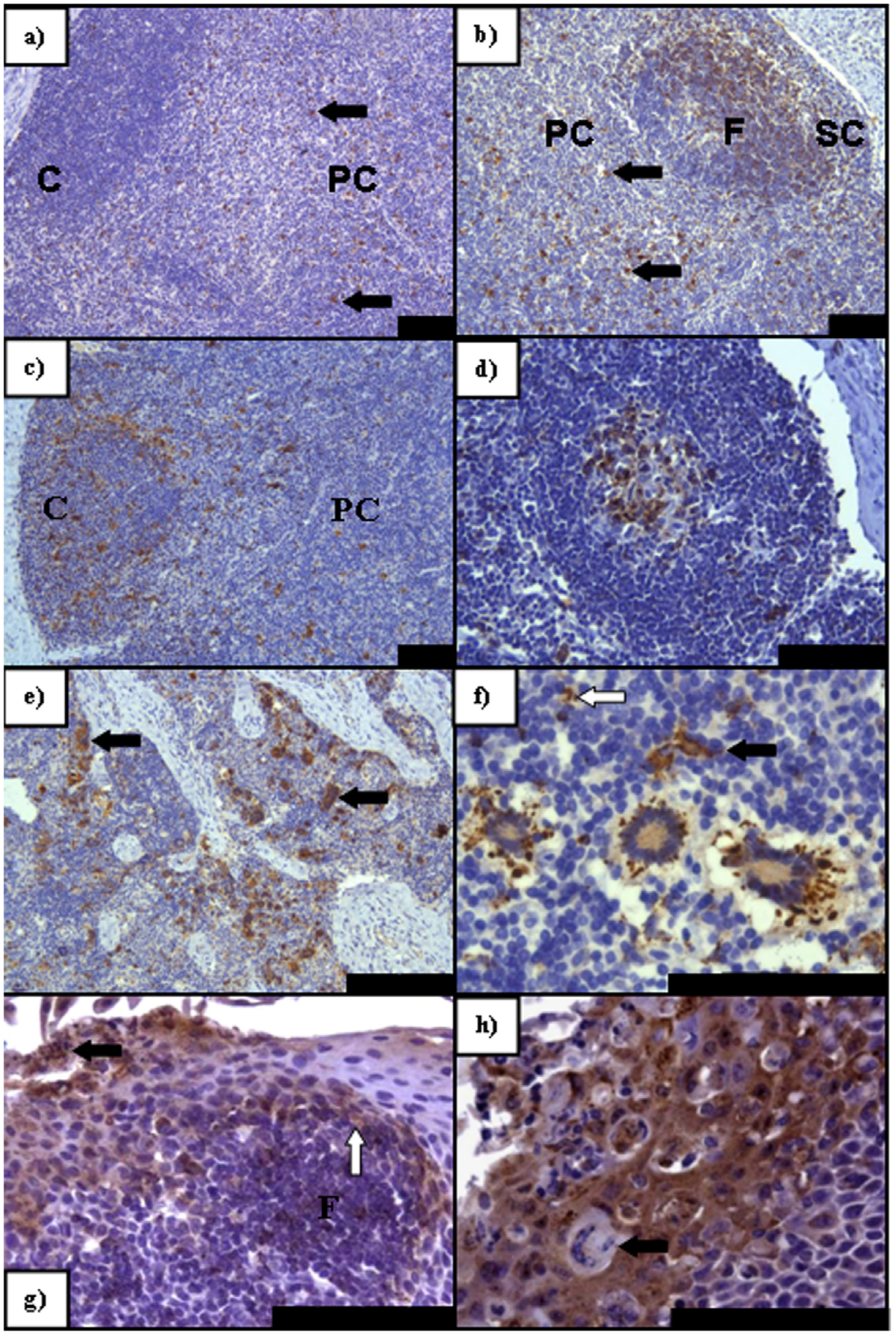
PPRV IHC on sections of lymphoid tissue taken at PME showing pertinent features of PPRV infection. a) A greater degree of immunolabelling (arrows) is seen in the paracortex (PC) (arrows) of the RPLN than in the cortex (C) (5 dpi); **b)** Antigen distribution in the subcapsular layer and the follicles at 7 dpi in the MLN. Paracortical virus antigen still remains (arrows); **c)** Primarily cortical immunolabelling within the RPLN (9 dpi) with antigen also remaining within the PC; **d)** In contrast to b) the germinal center of this follicle within the MLN contains virus antigen that is absent from the follicular mantle (9 dpi); **e)** Intense immunolabelling within the LPSLN medulla (5 dpi) with extensive syncytia formation (arrows); **f)** Predominately peripheral paracortical immunolabelling of syncytia within the LPSLN (7 dpi). Dendritic-type cells also present and positive for virus antigen (arrow) with an infected lymphocyte also present (open arrow); **g)** Immunolabelling within pharyngeal tonsil (5 dpi) indicating early epithelial infection noted both basally (open arrow) adjacent to an infected lymphoid follicle (F) and apically, abutting the crypt lumen (solid arrow); **h)** Advanced epithelial infection of the pharyngeal tonsil (7 dpi) with syncytia formation. All scale bars represent 100 µm.

Detection of virus antigen in lymphoid tissues is summarised in Table 3. Examples of antigen detection within lymphoid tissues at different time points post infection by IHC are shown in Figure 3. The earliest detection of PPRV by IHC was in samples taken at day 5 (Figure 3A, 3E and 3G). By HP on tissues taken 5 dpi, all lymph nodes assessed contained virus antigen to some degree with the exception of the follicle/mantle region (Table 3). Often, syncytia were present in the paracortex, medullary cords and in some regions of the non-follicular cortex and subcapsular area, with large numbers of necrotic and apoptotic cells (Figure 3A–C). Positive immunolabelling was seen in abundance in tonsillar sections and all sampled lymph nodes by IHC (Figure 3D–E). Generally, a paracortical response to the infection, consisting of larger lymphoblastic cells was seen, with multifocal areas of necrosis/apoptosis (Figure 3F). Hyperaemia was noted in most regions of nodes with margination of neutrophils observed. At 5 dpi changes within cortical primary follicles were minimal. Viral antigen distribution was similar at 7 dpi, although the regenerative response observed at 5 dpi appeared to have altered with dispersal of cells from the paracortex and medulla, and between cortical follicles, giving a depleted appearance. Syncytia were present throughout both the mantle and germinal centres of cortical follicles, though the level of lymphoid necrosis was reduced (Figure 3F). The retropharyngeal lymph node (RPLN) was seen to have the greatest immunolabelling intensity, with the tonsil exhibiting slightly higher levels of virus antigen than the other nodes, especially at 7–9 dpi (Table 3). A general increase in positive immunolabelling was observed in all lymphoid tissues at 7 dpi, followed by a slight decrease at 9 dpi. An exception to this rule was the mesenteric lymph node (MSLN), where viral antigen levels appeared to increase with disease progression. By 9 dpi the size and density of the cortex and cortical follicles was reduced with a recovery not being noted until 21 dpi, with a conspicuous plasma cell response observed in these sections.

A shift in antigen distribution was observed as infection progressed: The paracortical region of lymph nodes was most severely affected from 5 dpi (Figure 3A), before a discernible increase in cortical immunolabelling, accompanied by a decrease in antigen detection in the paracortex (Figure 3B and 3C). Viral antigen present in follicles increased markedly by 7 dpi (Figure 3B–D) with a shift in antigen detection from the follicular mantle to the germinal centres between 7 and 9 dpi. The detection of virus antigen in the medulla of lymph nodes generally resembled those seen in other parts of the node throughout the infection period (Figure 3E).

Cell types supporting infection also altered during the course of infection with early infection of cells having morphological characteristics of a dendritic or monocytic lineage. As infection progressed, lymphocyte infection increased. Interestingly, the tonsillar epithelium often contained large numbers of infected lymphocytes and reticular cells adjacent to apparent early infection of the basilar epithelium, especially in early sections taken at 5 dpi (Figure 3G). Less commonly, epithelial infection appeared independent of lymphoid involvement. Certainly, as infection progressed, epithelial infection was frequently marked and typified by antigen detection in the vast majority of cells, accompanied by a neutrophilic exudate, which did not label positively for virus antigen (Figure 3H). Splenic immunolabelling was seen at a very low level, increasing between 7 and 9 dpi. The strongest immunolabelling was seen in the peri-arteriolar lymphoid sheath area (PALS) consisting primarily of T lymphocytes, with lower numbers of positive lymphocytes noted in the red pulp (Table 3).

### Viral Antigen Distribution within Facial Epithelia

**Figure 4:**
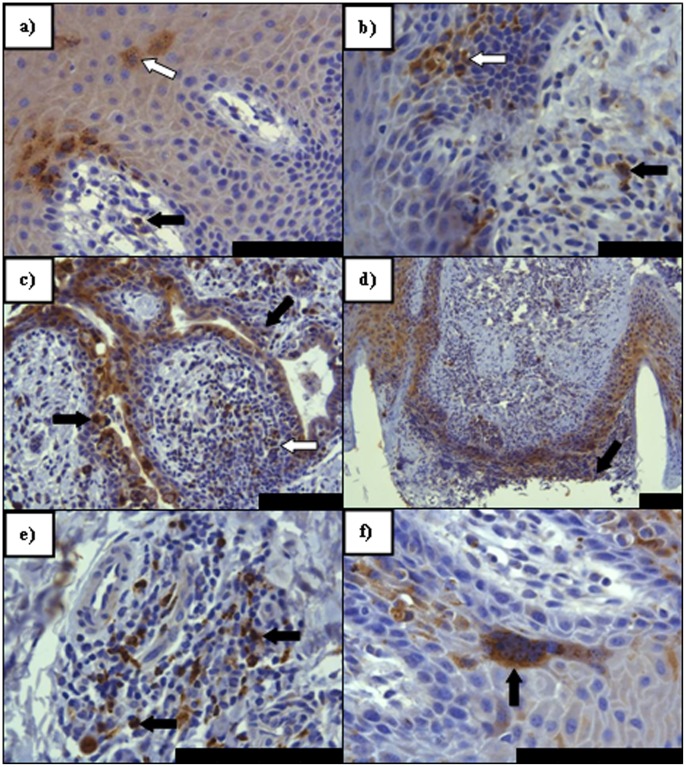
PPRV IHC on sections of facial epithelial tissue. a) Nasal mucosal epithelium (7 dpi). Immunolabelling of lymphoid (arrow) and epithelial cells (open arrow) indicating early PPRV infection of the nasal lamina propria (LP) and mucosal epithelium. Epithelial immunolabelling is noted almost exclusively in basal layers, surrounding LP papillae, with extension into the stratum spinosum; **b)** Lingual mucosal epithelium (9 dpi) Immunolabelling indicative of early infection in both the basal epithelium and stratum spinosum (arrow) alongside positively labelled immune cells in the LP (open arrow); **c)** Conjunctival mucosal epithelium (9 dpi). Evidence of advanced epithelial and proprial infection involving a mixed population of inflammatory and epithelial cells around an exocrine gland (arrows). Note in particular the immunolabelling within the proprial lymphoid follicle circumscribed by this gland (open arrow); **d)** Nasal skin (9 dpi) - marked epithelial infection and erosion (arrow) in and around two hair follicles; **e)** Labial mucosal epithelium (9 dpi) Following epithelial infection, lymphoid follicles were often seen to have formed in the LP of facial mucosae. Here a large lumber of positively immunolabelled lymphoid cells are seen (arrows); **f)** Labial mucosal epithelium (9 dpi) with a large epithelial syncytium (arrow) seen in the lower stratum spinosum layer. All scale bars represent 100 µm.

Detection of virus antigen in facial epithelia is summarised in (Table S2) whilst examples of antigen detection within these tissues at different time points post infection by IHC are shown in Figure 4. No virus antigen was detected in any of the tissues taken on day 2 and day 5 post infection by HP or IHC. Mucosal erosions in nasal sections were seen at 7 dpi, with mild epidermal cellular swelling and increased numbers of lymphocytes and reticular type cells seen migrating through the lamina propria and mucosa. IHC detected viral antigen within the epithelium and lymphoid cells of the lamina propria in nasal skin/mucosal samples at 7 dpi (Figure 4A) with some involvement of reticular-type cells in the conjunctival lamina propria of a single animal (Table S2). A mild, multifocal, acute, primarily lymphocytic inflammation of tongue and labial mucosa, with lower numbers of macrophages and neutrophils was seen by HP at 9 dpi alongside a similar multifocal epidermitis and folliculitis of haired skin of the lip, nasal bridge and eyelid, in some areas forming micro-abscesses. A slight increase in severity was noted multifocally in nasal sections at 9 dpi and inflammatory cell numbers and epithelial cell involvement in the nasal mucosa increased in the conjunctival mucosa, where large numbers of plasma cells were seen. Virus antigen was most commonly detected within the stratum spinosum layer of each epithelial tissue assessed (Table S2). The basal cell layers were sometimes found to be affected to a similar degree as the spinosum, but generally not in excess (Figure 4A–D). Interestingly, infected epithelial cells were detected in numerous hair follicles with concomitant infection of inflammatory cells such as macrophage/reticular cells and lymphocytes (Figure 4D). A greater antigen burden was detected in nasal, labial and conjunctival mucosal cell types at 9 dpi (Figure 4C–F) with antigen also being detected in the epithelium and proprial lymphoid tissues of the tongue (Figure 4B). Epithelial syncytia were not commonly seen although an example of a syncytium in the stratum spinosum of the labial epithelial is shown in Figure 4F. Typically epithelial infection was seen alongside lymphoid infection, though this was not an absolute rule, especially in some hair follicles. As with the lymphoid tissues, no evidence of ongoing infection could be demonstrated using IHC at 21 dpi.

### Virus Antigen Distribution within the Gastrointestinal (GI) Tract

**Figure 5:**
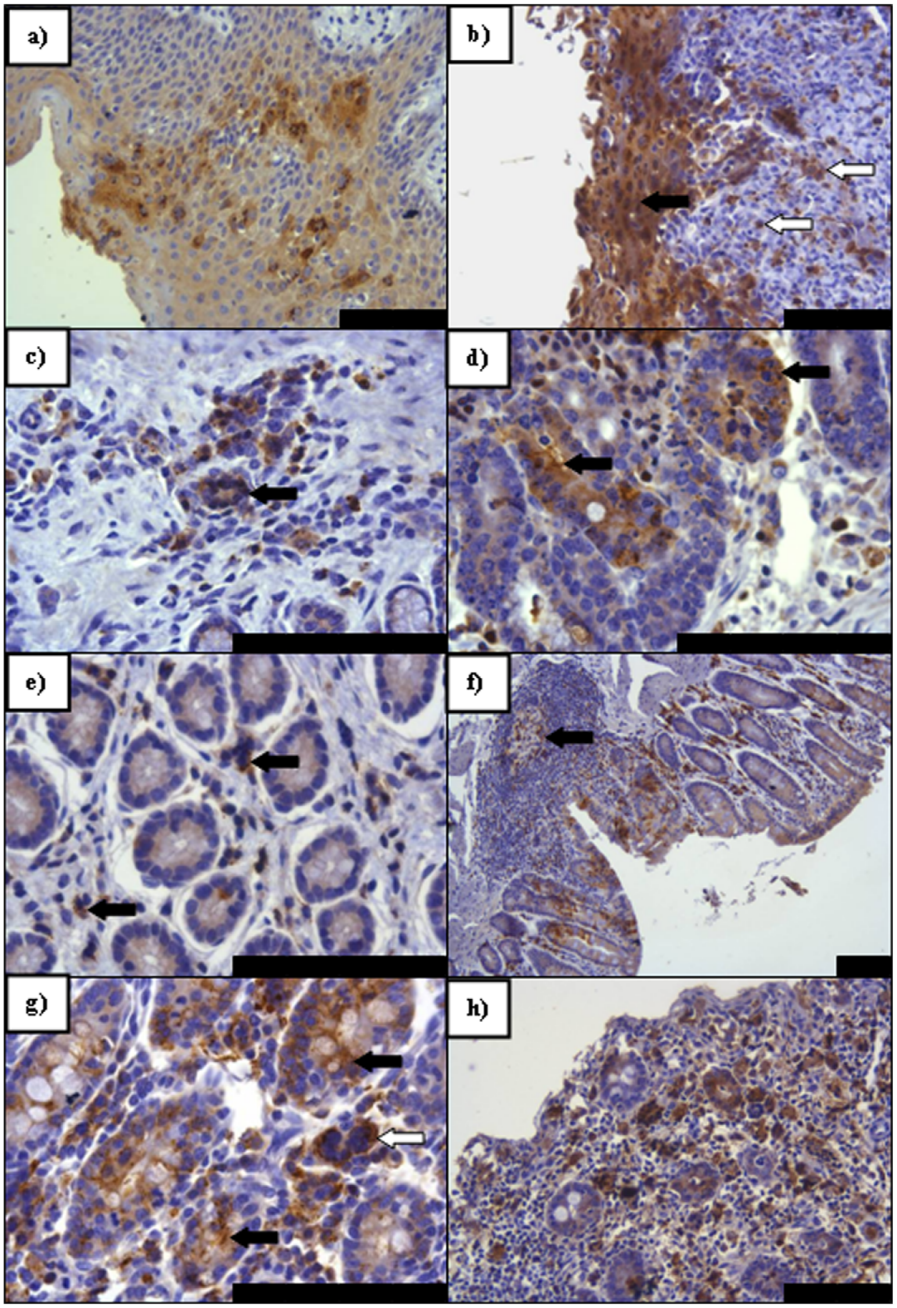
PPRV IHC on sections of digestive tract tissue taken at PME showing pertinent features of PPRV infection. a) An isolated focus of virus antigen detected in the omasum (7 dpi) in an area of epithelial trauma; **b)** Marked immunolabelling both of the epithelial (arrow) and proprial cells (open arrows) within the oesophagus (9 dpi); **c)** Foci of infection within abomasal crypts amongst lymphoid cells including a small syncytium (arrow); **d)** Severe abomasal infection (9 dpi) of crypt epithelial cells (arrows); **e)** Positively labelled lymphocytes (arrows) disperse throughout the lamina propria (LP) of the rectum (9 dpi); **f)** A lymphoid aggregate in rectal epithelia (9 dpi) taking the form of a true follicle with a germinal centre (arrow) containing many positively immunolabelled lymphocytes; **g)** Positive immunolabelling in the caecum (7 dpi) seen abundantly in proprial lymphocytes and within caecal glands (arrows) alongside a lymphoid syncytium (open arrow); **h)** Marked viral infection of both glandular epithelial cells and the immune/inflammatory cells present within the caecum (9 dpi). All scale bars represent 100 µm.

Examples of detection of virus antigen within the GI tract at different time points post infection by IHC are shown in Figure 5 whilst virus antigen detection in theses regions is summarised in (Table S3). By HP, incidental multifocal epithelial erosions with an apical neutrophilic exudate were seen at 9 dpi in sections of oesophagus and omasum with evidence of epithelial cell death, syncytia formation, sloughing of the upper keratinised layer and disruption of cellular architecture. In the adjacent lamina propria there was increased lymphoid infiltration, with occasional formation of lymphoid aggregates/follicles. Furthermore, throughout the intestines, from 7 dpi onwards and in the abomasum from 9 dpi, marked, diffuse lymphoid infiltration and oedema, causing varying levels of crypt disruption was observed. Lymphoid syncytial formation was seen within the lamina propria with associated cellular necrosis. Epithelial effects of PPRV infection were seen to a very small extent in the intestines at 7 dpi with pyknotic nuclear debris seen amidst normal appearing epithelial cells. This was also seen 9 dpi, where there was loss of cryptal architecture and cell death, occasionally leading to the formation of crypt abscesses. By IHC analysis, virus antigen within both epithelial and lymphoid structures were seen in the omasum of one animal euthanased at 7 dpi (Figure 5A). For the proximal organs of the gastrointestinal tract both epithelial and proprial lymphoid cells contained virus antigen at 9 dpi (Figure 5B–D). In such areas positively-labelled immune cells were more abundant with syncytia being present (Figure 5C, 5E and 5G). No virus antigen was detected in the reticulum of any animal.

In the early stages of intestinal involvement, foci of epithelial infection were usually seen in association with adjacent, positively immunolabelled leucocytes, which were also seen migrating within intestinal cryptal structures (Figure 5E). Clusters of positively immunolabelled epithelial cells, or severely affected crypts, were often found grouped around infected proprial lymphoid follicles (Figure 5F). Infection within mucosal epithelial cells appeared to initiate at mid/basal crypt level, extending in a luminal direction with time. The caecum appeared to be the most severely affected with extensive pathological lesions observed at 7 dpi, with an increase in both the severity of histopathological lesions and the level of viral antigen noted at 9 dpi, prior to the complete clearing of detectable viral antigen at 21 dpi (Figure 5G–H).

### Distribution of Virus Antigen in Other Tissues

No significant pathological changes were recognised in the urinary system, the hepatic parenchyma or the gallbladder. However, a primarily lymphocytic inflammatory response was observed by HP within the lamina propria of the gallbladder and rare syncytia were observed with multifocal erosion of neighbouring epithelium. By IHC, virus anigen was detected multifocally in the gall bladder epithelium and proprial leucocytes of one animal at 9 dpi, with proprial immunolabelling also present in a second animal. Within the respiratory system, the tracheal epithelium did not appear to be severely affected, though again a multifocal inflammatory cell infiltration of the epithelial layer was seen. Indeed, by IHC, tracheal sections were positive for viral antigen in both proprial immune cells and epithelial cells from 7 dpi. No effects attributable directly to PPRV infection were seen in the lower respiratory tract by HP or IHC. The heart also appeared unaffected, with the incidental finding of lipofuscin granules within the myocardial cytoplasm being the only abnormality detected. Peripheral blood vessels elsewhere did not appear to have been damaged post-infection. No virus antigen was demonstrated by IHC in the liver, kidney, urinary bladder or heart.

## Materials and Methods

### Virus stocks

The PPRV Côte d’Ivoire 89/1 strain (CI/89), a virulent field strain, was supplied in freeze-dried form, a kind gift from Dr Adama Diallo, of IAEA, Vienna, Austria. This original material was passaged once in lamb kidney (LK) cells, before storage at –80°C. Virus titre was assessed in tissue culture and was calculated as described previously [42].

### Experimental Inoculations

Eighteen indigenous female British white goats between 3 and 4 years of age were housed within the high security containment facility at the Pirbright Institute (formerly known as the Institute for Animal Health). All experimentation was performed in strict accordance with Home Office regulations (PPL 70/6212) and animal husbandry protocols following ethical acceptance at the Pirbright Institute, Pirbright Laboratory. Animals were monitored daily and a clinical score system was utilised to assess clinical progression and define humane endpoints. Goats were randomly allocated into two experimental groups housed within the isolation facility. All animals were monitored prior to inoculation to ensure they were in good health and establish baseline health parameters. Goats (n = 15) were infected intranasally dropwise with 500 µl PPRV Côte d’Ivoire 89/1 (CI/89), in MEM in each nostril (Total inocula, 10^4^ TCID_50_/animal) using a 2 ml syringe to deposit inoculum to both nostrils. To ensure that inocula remained within the turbinates the muzzle of each animal was held in an elevated position for 20 seconds following inoculation. Negative control animals (n = 3) received virus free media and were housed separately but within the same air space.

### Clinical and Post Mortem Sampling

Rectal temperatures were taken daily throughout the experimentation. Animals were examined daily for oral lesions and oculo-nasal discharge. All animals were scored for clinical disease and a clinical score template was developed for PPRV infection based on findings from this study and both published and unpublished observations (Table 1) utilising defined scales for healthy animals to guide baseline criteria [43], [44]. Behaviour, morbidity, appetite, nature of respiratory status, character of droppings and hydration levels were also recorded daily. All clinical sampling was completed by 12 pm on each sampling day to minimise any diurnal fluctuations in parameters. A sub-group of three goats were euthanased for full post mortem examination on days 2, 5, 7, 9 and 21 days post-inoculation (dpi). Post mortem samples taken are detailed in Table S1.

Heparinised and whole clotted blood samples were taken at the time of inoculation and at subsequent sample points on animals that remained at each time point following the schedule of euthanasia, 0 (n = 15), 2 (n = 12), 5 (n = 9), 7 (n = 6), 9 (n = 3), 12 (n = 3), 14 (n = 3), 16 (n = 3) and 21 (n = 3) days post-inoculation (dpi). Circulating peripheral blood leukocyte (PBL) levels were assessed prior to infection and averaged according to numbers seen at each of three pre-bleeds. Using this data as a baseline for each animal, the effect of infection on PBL levels was tracked following inoculation with PPRV CI/89. To assess virus excretion, conjunctival swabs were taken from the three inoculated animals that completed the study. All clinical samples were processed as described previously by Das et al., [45] and virus isolation from PBLs and eye swabs was attempted by co-cultivation with CHO-SLAM cells [46]. All post mortem tissues taken were trimmed to a size of no greater than 5 mm in the smallest dimension and no greater than 10 mm in the largest and were fixed in 40% formaldehyde (Surgipath) (100% formalin) diluted 1∶8 in phosphate-buffered saline (PBS) (Sigma). Tissues were left in fixative for 24 hours with the fixative solution being changed after 12 hours. Fixed samples were then embedded in paraffin wax and cut to 4 μ using a microtome (Leica RM2135) before being bound to Superfrost Plus slides (‘AnalR’, BDH) overnight.

### Molecular Detection

Molecular methods were performed as described previously [46]. Briefly, total cellular RNA was extracted from all samples taken to enable RT-PCR detection of viral nucleic acid. RNA was extracted using the TRIzol method as per the manufacturer’s instructions (GibcoBRL). Reverse transcription reactions were carried out using Superscript III (Invitrogen) and random hexanucleotide primers (RHPs). PCR detection of viral RNA in clinical samples (PBLs and eye swabs), was carried out using established PPRV specific primer sets F1b (5' AGTACAAAAGATTGCTGATCACAGT 3′, PPRV F gene, nucleotides (nt) 760>784) to F2d (5' GGGTCTCGAAGGCTAGGCCCGAATA 3′, PPRV F gene, nt 1183<1207), specific for the F gene [44] to generate a 448 base pair (bp) product. A nested PCR using internal primers F1 (5'-ATCACAGTGTTAAAGCCTGTAGA GG-3', PPRV F gene, nt 777–801) and F2 (5'-GAGACTGAGTTTGTGACCTACAAGC-3', PPRV F gene, nt 1148–1124) was also carried out, and where positives were detected, generated a product of 372 bp. RT-PCR controls were carried out using cellular β-actin (BA) specific primers: BA1 (5'-GAGAAGCTGTGCTA CGTCGC-3′, nt 152–171) and BA2 (5'-CCAGACAGCACTGTGTTGGC-3′, nt 395–414) to generate a 275 bp product.

### Serological Detection

The PPRV-specific antibody response was determined using the cH-ELISA as developed by Anderson et al., [47]. Serological results were expressed as a percentage inhibition (PI) of monoclonal antibody binding. The cut-off value between negative and positive serum was taken as 50% inhibition. PPRV-neutralisation assays were also performed as described by the OIE [41].

### Histopathological Examination of Post-mortem Tissues

Tissue sections were stained with haematoxylin and eosin for standard histopathological (HP) analysis using established techniques [48]. Material from uninfected animals was used as negative control tissue for both histopathological (HP) and immunohistochemical (IHC) techniques. For IHC, following deparaffinisation and rehydration of the tissue sections, slides were incubated for 30 minutes in Target Antigen Retrieval solution (DAKO), preheated to 95°C in a water bath and then cooled at room temperature for 30 minutes. Slides were fitted into the Sequenza coverplate system (Shandon). Known positive and negative (un-infected) tissue controls were included to assure the integrity of the primary antibody and procedure. A further negative control was implemented by running a parallel experiment using sample and control tissues, omitting the primary antibody. Sections were washed with 1x TBS (0.05 M, pH 7.6). A biotin blocking system (DAKO) was applied as per the manufacturer’s instructions prior to a 30 minute incubation step in 3% tryptone casein peptone (TCP) blocking solution. Viral antigen was labelled with RPV hyperimmune serum (RPVHIS) (Anderson et al., 1996) in a 1∶500 dilution in 3% TCP. Endogenous peroxidase activity was quenched with 3% hydrogen peroxide (Sigma) before a polyclonal swine anti-rabbit biotinylated immunoglobulin (DAKO. 1∶500 dilution in 3% TCP) was added. Samples were then incubated with streptavidin-horseradish peroxidase (S-HRP; diluted 1∶500), and the subsequent chromogenic reaction with added 3,3'-Diaminobenzidine (DAB+ Liquid, DAKO) was visualised as described previously [47].

## Discussion

The pathogenesis of PPRV is poorly defined, with the majority of knowledge coming from comparison with the closely related RPV [48]–[54]. Where PPRV pathogenesis has been examined, through either natural or experimental infection, assessment of pathomorphological disease progression has generally been studied during the later stages of disease where clinical features consistent with both RPV and PPRV infection have been well defined. Early events following infection have not been thoroughly examined for either natural infection or following vaccination with the current live attenuated vaccines. Here we sought to examine both the early events following virus infection and alongside this generate a clinical scoring system that can be utilised in future experimental inoculations with PPRV as well as for the assessment of natural outbreaks of PPR in the field. Whilst others have detailed a stepwise assessment of potentially infected animals for infection with PPRV [55], there is no clinical scoring system that enables disease symptoms to be graded and scored to enable ethical euthanasia of animals during late stage disease. Here, using data from the current study as well as outcomes of both published and unpublished experimental and natural infections we have generated a clinical score sheet to enable grading of the full spectrum of PPRV infection. Furthermore, using both molecular and immunohistochemical methods to detect both viral nucleic acid and antigen we have examined early and late events following infection.

The experimental inocula was a highly virulent field isolate of PPRV, the CI/89 strain [56]. Intranasal infection was used to mimic natural infection in contrast to previous experimental PPRV pathogenesis studies that have infected animals subcutaneously [16], [57], [58]. Surprisingly, considering the known virulence of the challenge material in the field, the infection resulted in mild clinical disease in our experimental animals. Despite this, both pyrexia and leucopaenia developed although severe disease did not occur. Typically for PPRV, fever develops 3–4 post inoculation and precedes the onset of clinical disease which may vary according to the strain inoculated, the route of inoculation and the immunological status of the infected animals. In this study 9 out of 12 inoculated animals (excluding those animals euthanased 2 dpi) developed a pyrexic response by 5 dpi and all 12 became pyrexic by day 7 dpi. Furthermore, 11/12 goats developed a marked leucopaenia by day 5, with a minimal reduction in circulating PBL counts of 25% when compared to circulating PBL populations from day 0.

Visible signs of mild clinical disease were observed within 3–4 days of the establishment of pyrexia. As disease progressed, mucoid nasal discharges, mucosal hyperaemia and brief anorexia were the main features. Mucosal erosions were not extensive and soft faeces, rather than frank diarrhoea was observed between 8–10 dpi, prior to a full clinical recovery. This disease course resembled the subacute form of PPRV [1] more closely than would be expected for this strain, especially following the acute onset of pyrexia and leucopaenia. Interestingly, despite the lack of clinical disease progression, viral replication appeared, through analysis of both swab material and post mortem tissues, to progress quickly following infection. It has been widely reported that following virus entry and replication, infection of lymphocytes serves to spread virus throughout the animal via both lymphatic and vascular systems. In this study, viral RNA was detected in both PBL and conjunctival samples from 5 dpi, in conjunction with the development of fever. PPRV is highly cell associated, as are all morbilliviruses [59], [60], and so it is assumed that virus reaches the conjunctiva within peripheral blood leucocytes, although autologous/heterologous infection of the conjunctiva by excreted virus produced in the nasopharyngeal cavity must also be considered. Infectious virus could not be demonstrated by virus isolation from conjunctival swabs until 7 dpi. However, detection of viral material by PCR at 5 dpi suggests that virus may be present earlier in lacrimal secretions. Similar observations have been reported previously where PPR viral RNA was been detected in nasal and ocular excretions prior to the appearance of visible clinical lesions in goats (57). This feature of infection may be of great significance to the transmission of virus to in-contact animals and further studies are required to assess the pre-clinical excretion of virus following infection.

The gross pathological findings were consistent with the mild nature of the clinical disease observed. An interesting finding was the difficulty in the detection of Peyer’s patches in the ileum of virus infected animals in the absence of obvious necrosis or haemorrhage in these areas. Previous studies have reported association of PPRV with these lymphoid structures and extensive necrosis and collapse of Peyer’s patches has been observed in both natural and experimental infections [16], [23], [26]. In contrast to infected animals, Peyer’s patches were readily detected in uninfected tissues of control animals which may imply that during infection a redistribution of lymphocytes from these aggregates to sites of infection occurs as suggested following CDV infection [36]. Histopathological assessment of infected tissues also highlighted common characteristics of morbillivirus infection (e.g., syncytial formation, and extensive necrosis) whilst other well defined features were absent (e.g., inclusion bodies). In particular, large numbers of syncytiated cells, ranging in viability and size were seen in the paracortex of lymph nodes from day 5 and in the cortex and follicles of lymph nodes, splenic white pulp, and gastrointestinal submucosal lymphoid tissue from day 7. Necrosis/apoptosis of syncytia and individual cells was marked in paracortical areas on day 5, but declined after this point, indicating that nuclear debris is degraded very quickly within the lymph nodes. In tonsillar and splenic tissues, whilst lympho-depletion is a characteristic of infection, cell necrosis as manifested by nuclear debris was not as prominent as that seen in the lymph nodes. This observation highlights an interesting feature of mucosal associated lymphoid tissues (MALT) whereby following antigenic stimulation lymphoid cells are retained within MALT primed for that antigen for approximately 24 hours [61]. This would serve to concentrate PPRV infected cells and, as a result, virus replication to one site. Squamous epithelial syncytia were also observed in tonsillar, facial and digestive tract epithelial tissues.

Information regarding the early stages of PPRV pathogenesis is scarce within published reports. Studies with a recombinant CDV, expressing eGFP, detected virus in lymphoid organs prior to infection of epithelial tissues in ferrets [35] although the earliest time point studied in this instance was 7 dpi. Further studies with either infection of macaques with an eGFP-expressing MeV or infection of cattle with a RPV expressing eGFP both detected fluorescence at by 6 and 7 dpi, respectively but neither study addressed earlier time points post inoculation [2], [37], [46]. The current literature postulates that for PPRV and other morbilliviruses, the initial replicative focus is within the naso-pharyngeal/respiratory epithelium [62]–[64], prior to infection of regional lymphoid organs, where a second round of replication occurs [59], [65]. Von Messling et al., [36] speculated that lymphocytes were the primary target for an initial massive burst of replication within the oral cavity and these disseminated the infection to distant organs. Studies by Farina et al., [66], postulated that the primary targets for MeV are SLAM positive monocytes, dendritic cells (DCs) and lymphocytes within the respiratory tract as initial virus entry is thought to occur via CD150/SLAM which is absent from the surface of epithelial cells. In the present study we assessed virus distribution during the prodromal period at 2 and 5 dpi. Whilst no viral antigen was detected in any tissues at 2 dpi, by 5 dpi there was a significant amount of viral antigen detected within lymphoid tissues, which also included nodes not involved in the drainage of the nasopharyngeal mucosa. It has been previously established that lymphocytes and DCs enter lymph nodes via either the afferent lymph or high endothelial venules (HEVs) [67], [68], which express a number of leucocytic adhesion molecules not normally found in quiescent endothelial cells, and play an important role in leucocyte homing mechanisms [69]. The prescapular lymph nodes drain the skin of the shoulder and caudal cervical region via their afferent lymphatics. The likely absence of any virus replication in the skin at this point following intranasal inoculation, and inability to detect virus antigen in non-lymphoid tissues early in infection, leads to the conclusion that virus reached these distant lymphoid tissues via the blood and HEVs. We postulate that DCs present in the intraepithelial space and the lamina propria of the respiratory mucosa take up PPR virions from the lumen of the respiratory tract and migrate to the T-cell–rich areas of local lymphoid organs including the tonsil from which virus enters circulation. This then implies that a productive infection has occurred within the first five days post inoculation, supported by PCR positivity of PBLs and conjunctival samples at 5 dpi, but from when this virus dissemination was initiated is difficult to pinpoint with the data and samples at our disposal. Of note, the apparent greater success of PCR from eye swab samples as judged by the need for nested PCR for PBL samples (Table 2) is unlikely to be due to a more advanced replication in conjunctival tissues at this stage, but more likely a consequence of increased efficiency of cell harvest methods. Certainly, the necessity for a second round reaction to detect viral nucleic acid in PBLs may reflect the efficiency of cell harvest methods alongside the reduced circulating cell number following the development of leucopenia and may also explain our inability to isolate live virus from PBLs post infection. Previous *in vivo* studies have reported viral antigen in PBLs of animals infected subcutaneously with RPV 1 to 2 dpi [46], [70] and in lymphoid organs as rapidly as 1 day following intranasal RPV and CDV challenge [71], [72]. In contrast, intranasal infection with CDV using a ferret model of pathogenesis did not corroborate these findings. Indeed, circulating viral antigen could not be detected at 3 dpi using FACS analysis although antigen was detected by 7 dpi [36]. Our findings are, however, in agreement with studies with MV whereby virus is postulated to enter the host at the alveolar level through infection of macrophages or DCs that then shuttle virus to lymph nodes where local amplification and subsequent systemic dissemination occurs [38]. Unlike experimental infection, where high doses of virus are used to challenge animals, the incubation period following natural infection is expected to be longer [16]. This is postulated to be due to a reduced viral load reaching lymphoid tissues, so following inoculation of a high dose of virus in an experimental setting it is plausible that virus would reach the node prior to day 3 post-challenge. Certainly, the inability to detect virus antigen within the nasal or oral epithelial and submucosal tissue layers sampled prior to 7 dpi reduces the likelihood that there is an initial primary replicative phase within these tissues. Instead, the results suggest that virus may be transported to secondary lymphoid organs and, as in the case of the RPLN, via both primary afferent lymph from facial tissues including the tonsils and lymph that had passed through other facial lymph nodes as well as HEV portals. This hypothesis may validate the detection of increased levels of virus antigen in this node compared to distant lymphoid organs (Table 3).

In this study, the distribution of PPRV antigen in animals showing clinical disease were generally in agreement with those described previously [11], [15], [16], [73] and in some cases the severity of the histopathological/IHC findings belied the mild nature of the clinical signs and gross pathology. However, late stage disease is often characterised by high levels of virus antigen in both lung and splenic tissues. Indeed, recent analyses of the pathogenesis of PPRV have shown severe pathology and immunohistochemical localisation of viral antigen during late stage disease within the spleen and within the lungs [22], [23]. Of significance to the current study was the almost complete lack of respiratory pathology. No incidental pathological findings like those seen in other epithelial organs were present in the lung sections examined. While some tracheal infection of both proprial leucocytes and epithelial cells was seen, the lungs were completely free of virus antigen. As the lung is regarded as an important target organ for most morbilliviruses, with the exception of RPV [12], [20], [72], [73], [74], these findings were unexpected. However, the infection was clinically mild and it is clear that morbillivirus infections of low virulence often replicate to a lower extent [52], [70]. Thus, the observation by von Messling et al., [36] that lung infection was a late event, occurring only in the face of a high viral load may hold true for PPRV. Alongside a lack of virus antigen detection in the lungs, the current study only detected low levels of virus antigen within splenic material, again reflecting the mild clinical disease seen during experimentation. Interestingly, other recent reports detailing experimental CDV and PPRV infections, demonstrate less antigen deposition in splenic material compared to other lymphoid organs [16], [35].

The reasons for the mild disease seen despite inoculation with a virulent strain of PPRV are unclear. However, previous studies have observed similar outcomes [74]. It is postulated that breed susceptibility may play an important role in the development of clinical disease although mechanisms that dictate this remain unclear and differential susceptibility has not been explored beyond isolated observations [58], [75]. Other factors such as nutritional status and co-infection with pre-existing parasitic organisms may also contribute to exacerbated disease courses that lead to high morbidity and mortality rates [58], [76]. Furthermore, whilst we have assessed virus antigen distribution on day 2 post infection, clearly further studies need to be performed to assess each day post infection to enable a conclusive evaluation of PPRV pathogenesis at early time points. In conclusion, we report an extensive investigation into the pathogenesis of PPRV *in vivo* and present a clinical scoring methodology that can be applied to grade further experimental and field cases of PPRV. We have demonstrated that the intranasal inoculation of PPRV mimics that seen following natural infection and hypothesise that the initial site for PPRV replication is not within the epithelial cells of the respiratory mucosa as has been previously reported, but is within the tonsillar tissue and lymph nodes draining the site of inoculation- a model similar to that postulated for other morbilliviruses.

## Supporting Information

Table S1 **Tissues taken at post-mortem examination for histopathological (HP) and immunohistochemical (IHC) analysis.**
(DOC)

Table S2 **Tissues were assessed and graded as described in** **Table 3** **.** Days 2, 5 and 21 are omitted as no virus antigen was observed at these time points in tissues analysed. (H) – Haired epithelium. Bracketed values for lingual tissues are the average of two goats as no immunolabelling was observed in the third of the cohort.
(DOC)

Table S3 **Tissues were assessed and graded as described in** **Table 3** **.** Days 2 and 5 are omitted for purposes of clarity as no immunolabelling was observed at these time points.
(DOCX)
