# Supplementary material for: Early Events following Experimental Infection with Peste-Des-Petits Ruminants Virus Suggest Immune Cell Targeting
Source: PLoS One. 2013 Feb 13;8(2):e55830. doi: 10.1371/journal.pone.0055830 (PMC3572172; doi:10.1371/journal.pone.0055830)
Supplement: Table S1 — Tissues taken at post-mortem examination for histopathological (HP) and immunohistochemical (IHC) analysis. (DOC) [file pone.0055830.s001.doc]

**SI Table 1 Tissues taken at post-mortem examination for histopathological (HP) and immunohistochemical (IHC) analysis**

| **Organ system** | **Organs sampled** |
| --- | --- |
| **Lymphoreticular organs** | Retropharyngeal lymph node (RPLN), palatine tonsil, mandibular lymph node (MLN), left/right prescapular lymph node (L/RPSLN), mesenteric lymph node (MSLN), spleen |
| **Facial epithelium** | Tongue, lip, eyelid skin/conjunctiva, |
| **Gastrointestinal (GI) system** | Oesophagus, rumen, reticulum, omasum, abomasums, duodenum, jejunum, ileum, caecum, colon, rectum, liver, gall bladder |
| **Respiratory system** | Nasal skin and mucosa, lung parenchyma, including small bronchi, trachea |
| **Urinary/cardiovascular system** | Kidney, urinary bladder/ heart |
