# Supplementary material for: Early Events following Experimental Infection with Peste-Des-Petits Ruminants Virus Suggest Immune Cell Targeting
Source: PLoS One. 2013 Feb 13;8(2):e55830. doi: 10.1371/journal.pone.0055830 (PMC3572172; doi:10.1371/journal.pone.0055830)
Supplement: Table S2 — Tissues were assessed and graded as described in Table 3 . Days 2, 5 and 21 are omitted as no virus antigen was observed at these time points in tissues analysed. (H) – Haired epithelium. Bracketed values for lingual tissues are the average of two goats as no immunolabelling was observed in the third of the cohort. (DOC) [file pone.0055830.s002.doc]

**Table S2 Antigen distribution within facial epithelial tissues taken on different days post inoculation following challenge with the CI/89 strain of PPRV**

|  | | Group Average Detected Viral Burden | |
| --- | --- | --- | --- |
|  | | Day 7 | Day 9 |
| **Nasal Epithelium** | Stratum Corneum | + | +/++ |
|  | Stratum Spinosum | + | ++ |
|  | Stratum Basale | + | +/++ |
|  | Sup. Lamina Propria | + | +/++ |
|  | Deep Lamina Propria | 0 | 0/+ |
|  | Stratum Corneum (H) | / | + |
|  | Stratum Spinosum (H) | / | +/++ |
|  | Stratum Basale (H) | / | + |
|  | Sup. Lamina Propria (H) | / | 0/+ |
|  | Deep Lamina Propria (H) | / | 0/+ |
| **Labial Epithelium** | Stratum Corneum | 0 | + |
|  | Stratum Spinosum | 0 | +/++ |
|  | Stratum Basale | 0 | + |
|  | Sup. Lamina Propria | 0 | + |
|  | Deep Lamina Propria | 0 | 0/+ |
|  | Stratum Corneum (H) | 0 | 0/+ |
|  | Stratum Spinosum (H) | 0 | + |
|  | Stratum Basale (H) | 0 | + |
|  | Sup. Lamina Propria (H) | 0 | 0/+ |
|  | Deep Lamina Propria (H) | 0 | 0/+ |
| **Lingual Epithelium** | Stratum Corneum | 0 | + (+/++) |
|  | Stratum Spinosum | 0 | +/++ (++) |
|  | Stratum Basale | 0 | + (++) |
|  | Sup. Lamina Propria | 0 | + (+/++) |
|  | Deep Lamina Propria | 0 | + (++) |
| **Conjunctival Epithelium** | Conj. Epithelium | 0 | +/++ |
|  | Lamina Propria | 0/+ | ++ |
|  | Haired Epithelium | 0 | + |
|  | Dermis | 0 | + |
