# Supplementary material for: Early Events following Experimental Infection with Peste-Des-Petits Ruminants Virus Suggest Immune Cell Targeting
Source: PLoS One. 2013 Feb 13;8(2):e55830. doi: 10.1371/journal.pone.0055830 (PMC3572172; doi:10.1371/journal.pone.0055830)
Supplement: Table S3 — Tissues were assessed and graded as described in Table 3 . Days 2 and 5 are omitted for purposes of clarity as no immunolabelling was observed at these time points. (DOCX) [file pone.0055830.s003.docx]

**Table S3 Antigen distribution within gastrointestinal tissues taken on different days post inoculation following challenge with the CI/89 strain of PPRV**

|  | | Group Average Detected Viral Burden | |
| --- | --- | --- | --- |
|  |  | Day 7 | Day 9 |
| Oesophagus | Stratum Corneum | 0 | + |
|  | Stratum Spinosum | 0 | + |
|  | Stratum Basale | 0 | + |
|  | Sup. Lamina Propria | 0 | + |
|  | Deep Lamina Propria | 0 | + |
| Rumen | Stratum Corneum | 0 | 0/+ |
|  | Stratum Spinosum | 0 | + |
|  | Stratum Basale | 0 | + |
|  | Sup. Lamina Propria | 0 | + |
|  | Deep Lamina Propria | 0 | / |
| Omasum | Stratum Corneum | 0/+ | + |
|  | Stratum Spinosum | 0/+ | +/++ |
|  | Stratum Basale | 0/+ | + |
|  | Sup. Lamina Propria | 0/+ | 0/+ |
|  | Deep Lamina Propria | 0 | 0/+ |
| Abomasum | Epithelium | 0 | + |
|  | Lamina Propria | 0 | ++ |
| Duodenum | Epithelium | 0/+ | +/++ |
|  | Lamina Propria | ++ | ++ |
| Jejunum | Epithelium | 0/+ | 0/+ |
|  | Lamina Propria | +/++ | +/++ |
| Ileum | Epithelium | 0/+ | 0/+ |
|  | Lamina Propria | +/++ | +/++ |
| Caecum | Epithelium | + | ++ |
|  | Lamina Propria | +/++ | ++/+++ |
| Colon | Epithelium | + | +/++ |
|  | Lamina Propria | +/++ | ++/+++ |
| Rectum | Epithelium | 0/+ | +/++ |
|  | Lamina Propria | +/++ | ++/+++ |
